# Mathematical Prostate Cancer Evolution: Effect of Immunotherapy Based on Controlled Vaccination Strategy

**DOI:** 10.1155/2020/7970265

**Published:** 2020-01-13

**Authors:** Dorota Ba̧dziul, Paweł Jakubczyk, Levan Chotorlishvili, Zaza Toklikishvilie, Julian Traciak, Joanna Jakubowicz-Gil, Sylwia Chmiel-Szajner

**Affiliations:** ^1^Medical Faculty, University of Rzeszów, Rejtana 16A, 35-959 Rzeszów, Poland; ^2^Faculty of Mathematics and Natural Sciences, University of Rzeszów, Rejtana 16A, 35-959 Rzeszów, Poland; ^3^Institute of Physics, Martin-Luther University Halle-Wittenberg, D-06099 Halle, Germany; ^4^Department of Physics, Tbilisi State University, Chavchavadze av. 3, 0128 Tbilisi, Georgia; ^5^Faculty of Mathematics and Applied Physics, Rzeszów University of Technology, Al. Powstańców Warszawy 12, 35-959 Rzeszów, Poland; ^6^Institute of Biology and Biochemistry, Maria Curie-Skłodowska University, Plac Marii Curie-Skłodowskiej 5, 20-031 Lublin, Poland

## Abstract

Basic immunology research over several decades has led to an improved understanding of tumour recognition by components of the immune system and mechanism of tumour evasion from immune detection. These findings have ultimately led to creating antitumour immunotherapies in patients with different kind of cancer including prostate cancer. The increasing number of reports confirms that immune-based therapies have clinical benefit in patients with prostate cancer with potentially less toxicity in comparison with traditional systemic treatments including surgical resection, chemotherapy, or radiotherapy in various forms. This review focuses on the possibility of modulation of the optimal immunotherapy based on vaccination strategy adopted to individual patients in order to increase quality and quantity of their life.

## 1. Introduction

Today, there are a number of immune-based cancer treatments in development for prostate cancer (PC). In papers [1, 2], it has been shown that the rate of increase of the prostate specific antigen (PSA) can be modified in terms of vaccinations that stimulate the patients' immune system. If it is supposed that PSA level can be treated as marker for disease load in PC, then one can build a model that describes mathematically appropriately adjusted immunotherapy to treat PC [2, 3]. Using such a model, one can shown that the efficacy of immunotherapy can be improved by changing the interdosing intervals rather than the dose itself [4, 5]. These facts suggest that, in order to achieve the best prostate cancer treatment, an optimal vaccination strategy matched to individual patients should be found [6, 7]. In this article, we try to develop a methodology that helps to find personalized vaccinations schedule that is optimal for treated patient based on mathematical model of PC immunotherapy developed by Kronik et al. in [8]. Our proposed vaccination schedule is taking into account the personal dynamics of the immune system and the rate of disease processes. Based on these data, it may be possible to better understand the field of therapeutic cancer vaccines.

The paper is organized as follows. At first, we briefly present the mathematical model of prostate cancer immunotherapy which we use and then discuss possible phenotypes that arise from this numerical model. Finally, we analyse chosen vaccination strategy on the population of statistically generated patients.

## 2. Material and Methods

The model of PC immunotherapy is represented mathematically by the system of seven ordinary differential equations that describe this very sophisticated biological process in simple form [8]. This simplification, however, is made in such a way that does not destroy the nature of the process and all important mechanisms are maintained. These equations can be analysed mathematically (cf. e.g., [9]) giving very useful information about the original process and allowing making prediction about the development of the disease. The model describes dynamical dependencies between the cellular vaccine (*V*) and prostate cancer cells (*P*) by the use of immune system, that is, antigen-presenting dermal dendritic cells (*D*_*m*_), mature dendritic cells (*D*_*C*_), “exhausted” dendritic cells (*D*_*R*_), antigen specific effector cells (*C*), and regulatory/inhibitory cells (*R*). At the cellular level, it can be presented as (1)–(7).

(1) $$\begin{array}{c} \frac{\text{d}V}{\text{d}t}=-k_{i}n_{\nu }V, \end{array}$$

(2) $$\begin{array}{c} \frac{\text{d}D_{m}}{\text{d}t}=k_{i}\left(V+V_{p}\right)-k_{m}D_{m}, \end{array}$$

(3) $$\begin{array}{c} \frac{\text{d}D_{C}}{\text{d}t}=\alpha_{l}k_{m}D_{m}-k_{CR}D_{C}, \end{array}$$

(4) $$\begin{array}{c} \frac{\text{d}D_{R}}{\text{d}t}=k_{CR}D_{C}-\mu_{D}D_{R}, \end{array}$$

(5) $$\begin{array}{c} \frac{\text{d}C}{\text{d}t}=a_{C}D_{C}-\mu_{C}C-k_{R}CR, \end{array}$$

(6) $$\begin{array}{c} \frac{\text{d}R}{\text{d}t}=a_{R}D_{R}-\mu_{R}R, \end{array}$$

(7) $$\begin{array}{c} \frac{\text{d}P}{\text{d}t}=rP-a_{p}CP\frac{h_{p}}{h_{P}+P}. \end{array}$$

Following [8] in Table 1 we present model parameters together with their definitions and units. See, for example, [8, 10, 11] for detailed information about model assumption, parameters description and evaluation, and biological mechanism presentation. Initial conditions of the model are given by estimation of the three types of cell populations in the starting point of the simulation for a patient with PC and without immunity. Standard dose of the vaccine contains 2.4 · 10^7^ cells. Therefore, with *V*(0), we take 2.4 · 10^7^ cells or the multiple of this. The initial populations of the immune system cells are taken to be equal to zero, so that *D*_*m*_(0)=0, *D*_*C*_(0)=0, *D*_*R*_(0)=0, *C*(0)=0, *R*(0)=0. Initial population of tumour cells is proportional to the tumour size and average tumour in the initial stage of PC contains about 30 · 10^9^ PC cells.

In order to determine which parameters contribute the most to output variability, we applied global quantitative sensibility analysis based on Sobol' (variance decomposition) approach [12, 13]. Sobol' global analysis is changing all of the model parameters at the same time and allows discriminating between insensitive and sensitive model parameters [13]. Moreover, this approach is proven to be one of the most robust and effective tools to describe individual and cooperative sensitivities. It gives indices that can be used for estimating the influence of individual parameters or groups of parameters on the model output. These sensibility indices allow deciding if the model output is sensitive to a chosen input parameter. If the output of the model is not sensitive to an input parameter, then the effect of that parameter can be neglected and it can be fixed, hence reducing the complexity of the model.

**Figure 1:**
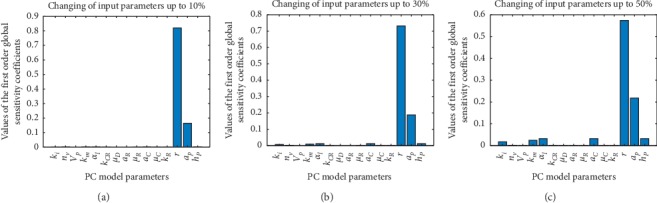
The graphs show the values of the first order global sensitivity indices for different ranges of variation of the input parameters. As we see, the greater the variability of the input parameters, the larger the indices for the input parameters except for *r* and *a*_*p*_, but, even for 50% of variability of the input parameters, the *r* and *a*_*p*_ indices are much more bigger than the rest of input parameters. (a) Δ = 10%, (b) Δ = 30%, and (c) Δ = 50%.

To make calculation, we have established time of the simulation, variation of the input parameters Δ, and the number of samples for the quasi-random Monte Carlo simulation. One has noticed that calculations of the sensitivity indices are independent of the time of the simulation and the number of samples for the simulation; therefore, we have performed calculations only for different variations of the input parameters Δ. We have calculated the parameters sensitivities of a PC immunotherapy model presented in equations (1)–(7)) based on Sobol' probability distribution function. The results of calculations are presented in Figure 1.

According to Figure 1, one can conclude that there are only two sensitive parameters *r* and *a*_*p*_ out of 15 input parameters (see Table 1) which differ for various patients; they are personalized. This corresponds to different clinical outcomes for patients and allows classifying them in terms of these parameters. Such approach leads to the personalized model of PC where patients generally differ from each other by four variables:

- Dose of the vaccine *V*(0)
- Initial tumour size *P*(0)
- Tumour growth rate *r*
- Maximal PC cell killing efficacy *a*_*p*_

The simulations of the PC model were performed on a Matlab Simulink environment (MathWorks, Natick, MA). We solved the model equations by implementing them in Simulink using graphical programming language, whereas strategy of calculation and parameters setting were implemented in the appropriate m-files. Meanwhile, to calculate the sensitivity indices (first order, global), we used the Matlab software tool GSAT (Global Sensitivity Analysis Toolbox) [14].

## 3. Phenotypes: Numerical Classification of Patients

Our methodology of research is based on the mathematical model of prostate cancer described in Section 2. The model allows calculating the number of PC cells at any time of the PC evolution. Taking into account the number of PC cells, one can easily calculate the volume of cancerous PSA-producing cells and then the rate of change of PSA [15].

**Figure 2:**
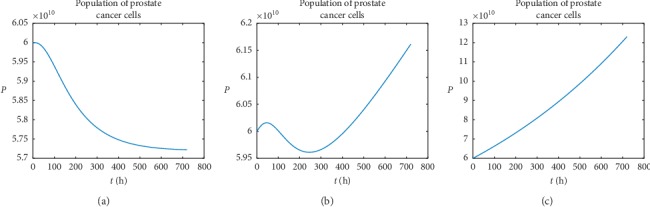
The graphs show examples of PC cells evolution for the three distinguished phenotypes. One can notice that, in the first case (a), patients are responding to immunotherapy, and in the second (b), they are poorly responding, while in the third (c), they are not responding at all. Here, *P*(*t*) denotes the population of PC cells over the time. (a) Phenotype I, (b) phenotype II, and (c) phenotype III.

We start numerical analysis of PC evolution lasting over a period of one month for the case of single vaccination. In this case, one can distinguish numerically only three possible phenotypes of PC developing. In the first phenotype, a statistical patient is clinically responding; he starts responding immediately after vaccination and maintains this state over some period of time. The example of such patient with personalized parameters given in Table 2 (row 2) is presented in Figure 2(a).

In the second phenotype, a statistical patient is delayed nonresponder. It means that the population of PC cell counts initially increases and then after two or three days decreases but finally rebounds. The example of such patient with personalized parameters given in Table 2 (row 3) is presented in Figure 2(b).

In the third phenotype, a patient is clinically nonresponding; the population of PC cell is increasing over the considered period of evolution and is independent from vaccination. The example of such patient with personalized parameters given in Table 2 (row 4) is presented in Figure 2(c).

**Figure 3:**
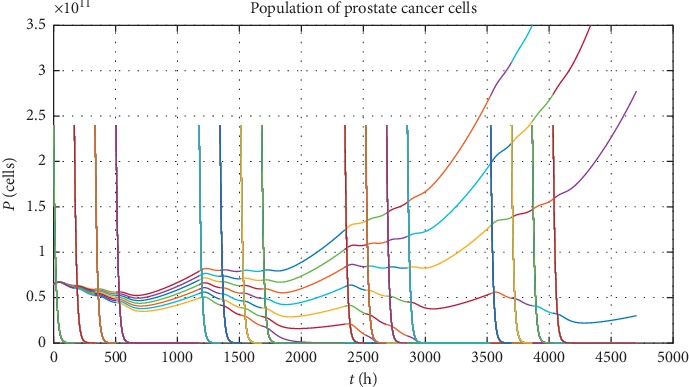
The possible scenarios of PC evolution for different maximal PC cell killing efficiency *a*_*p*_. Each line represents virtual patient labelled by *a*_*p*_ parameter. Quasi-vertical lines show the moments of vaccinations, while their heights correspond to the population of vaccinations cells rescaled by 10^4^.

**Figure 4:**
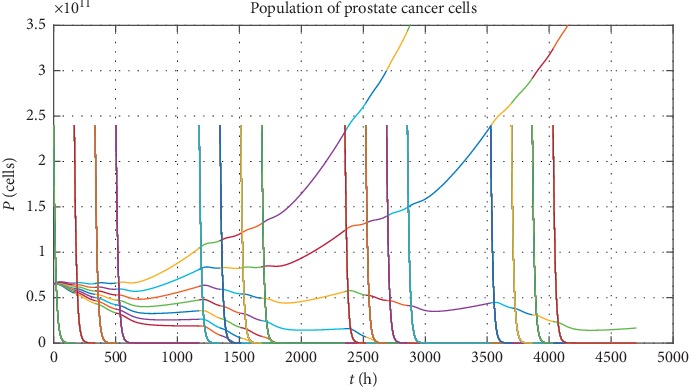
The possible scenarios of PC evolution for different tumour growth rate *r*. Each line represents virtual patient labelled by *r* parameter. Quasi-vertical lines show the moments of vaccinations, while their heights correspond to the population of vaccinations cells rescaled by 10^4^.

Next, in order to show different types of PC evolution, let us analyse numerically the chosen schedule of vaccinations. This vaccination schedule consists of a series of vaccinations lasting over half a year according to the timetable: one vaccination in a week over four weeks, with the next four weeks as a break for recovery, and repeating this scheme four times (see quasi-vertical lines on Figures 3 and 4 which represent the vaccinations). The results of such approach for 7 statistically chosen patients classified by *a*_*p*_ parameter, that is, *a*_*P*_=[0.310, 0.322, 0.333, 0.345, 0.357, 0.368, 0.380] · (10^−6^) (for each *a*_*p*_, we have *P*(0)=65 · 10^9^, *r*=10^−3^, and *V*(0)=2.4 · 10^7^), are presented in Figure 3. The *a*_*p*_ parameters are chosen in such way to show different evolution scenarios of the PC for maximal value of tumour growth rate *r* and to catch the moment when PC cells population starts to grow in an uncontrolled way. In Figure 3, each quasi-vertical line represents the vaccination, so that we have four cycles by four vaccinations each. One quasi-vertical line represents the population of vaccination cells rescaled by 10^4^ (i.e., vaccination cells = value form the graph divided by 10^4^). Seven coloured lines represent PC evolution for different patients, whereas changing of colour in each line is caused by vaccination. From the other side, we can choose patients (labelled by *r* parameter) with the same maximal PC cell killing efficiency *a*_*p*_ parameter and different tumour growth rate *r*, according to the list *r*=[0.01, 0.175, 0.34, 0.505, 0.67, 0.835, 1] · (10^−3^) (for each *r*, we have *P*(0)=65 · 10^9^, *a*_*p*_=0.25 · 10^−6^, and *V*(0)=2.4 · 10^7^). Then one can also observe the situation when population of PC cells grows in an uncontrolled way (see Figure 4).

This simple approach shows that, in order to make full analysis of PC evolution, we should vary *a*_*p*_ and *r* parameters together with the initial tumour size *P*(0) and the dose of vaccination *V*(0). Such complex analysis for virtual patients generated as points uniformly distributed in the whole space of accessible parameters is given in Section 4.

## 4. Analysis of Chosen Vaccination Strategies

In this analysis, we restrict ourselves to the three sizes of tumour: *P*_01_=30 · 10^9^, *P*_02_=45 · 10^9^, *P*_03_=60 · 10^9^[cells]. A group of 10100 patients has been generated for each size of tumour based on the scope of variation of *r* and *a*_*p*_. Every patient in that group has unique parameters of tumour growth rate (*r*[h^−1^]) in the range from 10^−5^[h^−1^] to 10^−3^[h^−1^] and maximal PCa cell killing efficacy (*a*_*p*_[cell^−1^ · h^−1^]) ranging from 0 to 2 · 10^−6^[cell^−1^ · h^−1^].

The treatment plan consisted of administering the dose of vaccine at weekly intervals for four weeks. The standard dose of the vaccine is 2.4 · 10^7^[cells] (1.0 d), and, in addition, half of standard dose (0.5 d) and one and a half of the standard dose (1.5 d) are given to the groups. After simulating the treatment, it can be concluded that, in many cases, the plan used quickly dealt with cancer in a large number of cases.

**Figure 5:**
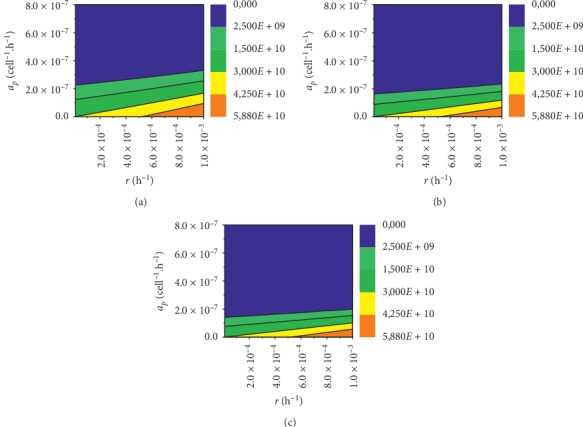
Contour plot of *a*_*p*_(*r*) dependency for tumour size of 30 · 10^9^ and three different doses of vaccine. (a) 0.5 dose, (b) 1.0 dose, and (c) 1.5 dose.

For tumour size of 30 · 10^9^ and for dosing of 0.5 d, vaccination has not completely cured the tumour for 1574 patients; additionally, for this group, vaccination did not help at all 472 patients, meaning that *P* > *P*_0_. Dependency *a*_*p*_(*r*) for given tumour size and dosing is presented in Figure 5(a). For dosing of 1.0 d, vaccination has not fully cured 1136 patients, 350of whom it did not help at all. Data for this group are presented in Figure 5(b). For dosing of 1.5 d, vaccination has not completely cured 986 patients, and from this group vaccination did not help at all 308 patients. Dependency *a*_*p*_(*r*) for given tumour size and 1.5 d dosing is presented in Figure 5(c).

**Figure 6:**
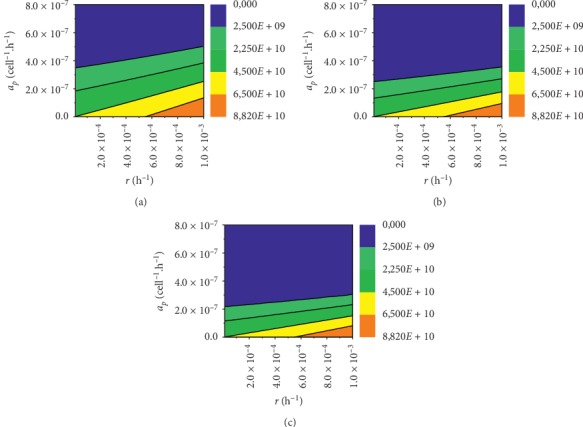
Contour plot of *a*_*p*_(*r*) dependency for tumour size of 45 · 10^9^ and three different doses of vaccine. (a) 0.5 dose, (b) 1.0 dose, and (c) 1.5 dose.

For the tumour size of 45 · 10^9^ and dosing of 0.5 d, vaccination has not completely cured the tumour for 2294 patients, of whom 681 were not helped at all by vaccination. Correlation *a*_*p*_(*r*) for given tumour size and dosing is presented in Figure 6(a). For 1.0 d dosing, vaccination did not cure tumour for 1659 patients, and from this group vaccination did not help at all 499 patients. Data are presented in Figure 6(b). Dosage of 1.5 d did not cure the tumour completely for 1430 patients, of whom 681 were not helped at all by vaccination. Data are presented in Figure 6(c).

**Figure 7:**
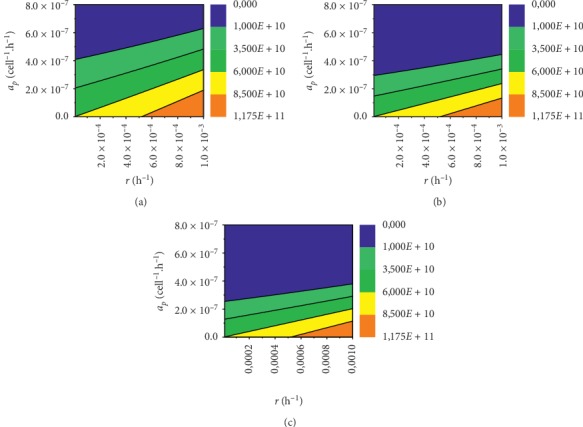
Contour plot of *a*_*p*_(*r*) dependency for tumour size of 60 · 10^9^ and three different doses of vaccine. (a) 0.5 dose, (b) 1.0 dose, and (c) 1.5 dose.

For the tumour size of 60 · 10^9^ and dosing of 0.5 d (Figure 7(a)), vaccination did not cure the tumour completely for 3014 patients, and from this group vaccination did not help at all 890 patients. Dosage of 1.0 d (Figure 7(b)) did not cure the tumour completely for 2170 patients, of whom 649 were not helped at all by vaccination. For dosing of 1.5 d (Figure 7(c)), vaccination did not cure the tumour completely for 1869 patients, of whom 565 were not helped at all.

With the assumed treatment model, the success of the treatment is visible already for very small *a*_*p*_ values. For a maximum tumour growth rate of *r*=10^−3^, the 30 · 10^9^ tumour disappears at *a*_*p*_=3.4 · 10^−7^[cell^−1^ · h^−1^] for dosing of 0.5 d and *a*_*p*_=2.4 · 10^−7^[cell^−1^ · h^−1^] for dosing of 1.0 d, and, for dosing of 1.5 d, tumour disappears at *a*_*p*_=2 · 10^−7^[cell^−1^ · h^−1^].

The tumour of size 45 · 10^9^ disappears at *a*_*p*_=5.2 · 10^−7^[cell^−1^ · h^−1^] and dosing of 0.5 d. For dosing of 1.0 d, tumour disappears at *a*_*p*_=3.6 · 10^−7^[cell^−1^ · h^−1^] and for dosing of 1.5 d tumour disappears at *a*_*p*_=3 · 10^−7^[cell^−1^ · h^−1^].

The largest tumour of size of 60 · 10^9^ is cured at *a*_*p*_=6.8 · 10^−7^[cell^−1^ · h^−1^] and dosing of 0.5 d. For dosing of 1.0 d largest tumour disappears at *a*_*p*_=4.8 · 10^−7^[cell^−1^ · h^−1^] and for dosing of 1.5 d tumour disappears at *a*_*p*_=4 · 10^−7^[cell^−1^ · h^−1^]. It can be noted that a multitude of vaccine doses affect the curability but not much. It can also be noted that very small changes in *a*_*p*_ cause very large changes in the number of cancer cells *P*. This trend can be observed in Figures 5–7.

## 5. Conclusion

Modern therapies, commonly used against cancer, still do not cure patients with advanced cancers. However, in contrast to chemotherapy or radiotherapy, personalized therapy extends the time free from symptoms of the disease, increases the quality of life of patients, which often allows them to return to work and full physical capacity, usually has minor side effects, and, most importantly, lengthens the patients' life time. Today, there are a number of immune-based treatments in development for different kind of cancer [16]. Immunotherapy for PC has spread and become resistant to other treatments and is a rapidly emerging approach to treatment. It relies on the ability of the immune system to identify and destroy tumour cells and to elicit a long-lasting memory of this interaction. Under ordinary circumstances, however, the ability of tumour cells to trigger an effective immune response is limited [17]. Understanding the dynamic relationship between cancer cells and the immune system increases the potential for developing new therapies that help improve the outcomes for patients with prostate cancer.

The purpose of this study was to determine if it is possible to make and administer safely a “personalized” vaccine to treat patients that have been diagnosed with prostate cancer and are not candidates for curative therapy. We have taken into consideration three hypothetical models of patients. The simulations carried out consisted in comparing the severity of the patient's disease and its response to the standard vaccination schedule with its modifications. We have shown that in the considered model from the numerical point of view there are only three possible phenotypes concerning PC evolution. Moreover, taking into account the results of the treatment of statistical patients, the proposed strategy of therapy is a promising one. It can be noted that, for unhealed groups of patients, over half of the cases show tumour responsiveness. Lack of responsiveness can only be noticed at low *a*_*p*_ values. Conducted analysis showed that immune responses against cancer are highly heterogeneous, not only between the level of cancer advancement but also within different patients with the same type of cancer, indicating that personalized immunotherapy should be employed, based on the immune status of the individual patient.

## Figures and Tables

**Table 1:** Parameters of the PC mathematical model. According to this model, only two parameters *r* and *a*_*P*_ are personalized and depend on the immunology system of the patients.

	Definition	Value	Units
*k*_*i*_	Rate of DC maturation following vaccine uptake	0.06	h^−1^
*n*_*ν*_	Number of vaccine cells required to induce maturation of one DC	1	—
*V*_*p*_	Natural influx of mature DCs	0	cells
*k*_*m*_	Rate of DC migration from skin to lymph node	0.027	h^−1^
*α*_*l*_	Fraction of antigen-presenting DCs entering the lymph node	0.03	–
*k*_*CR*_	Rate of exhaustion of mature DCs	0.027	h^−1^
*μ*_*D*_	Death rate of exhausted DCs	0.014	h^−1^
*a*_*R*_	Rate of inhibitory cell recruitment by exhausted DCs	3 · 10^−3^	h^−1^
*μ*_*R*_	Death rate of inhibitory cells	0.03	h^−1^
*a*_*C*_	Rate of effector cell recruitment by mature DCs	0.38	h^−1^
*μ*_*C*_	Effector cell death rate	0.007	h^−1^
*k*_*R*_	Rate of effector cell inactivation by inhibitory cells	6 · 10^−7^	cell^−1^ · h^−1^
*r*	Tumour growth rate	Personalized; range from 10^−5^ to 10^−3^	h^−1^
*a*_*p*_	Maximal PCa cell killing efficacy	Personalized; range from 0 to 2 · 10^−6^	cell^−1^ · h^−1^
*h*_*P*_	Effector cell efficacy damping coefficient	10^8^	Cells

**Table 2:** Example of PC model parameters corresponding to responder patient—phenotype I (row 2), delayed nonresponder patient—phenotype II (row 3), and nonresponder patient—phenotype III (row 4).

Phenotype	Initial number of PC cells *P*(0)	Tumour growth rate *r*	Maximal PCa cell killing efficiency *a*_*p*_	Dose of vaccination *V*(0)
I	60 · 10^9^	0.1 · 10^−5^	2 · 10^−6^	2.4 · 10^7^
II	60 · 10^9^	0.99 · 10^−4^	1.9 · 10^−6^	2.4 · 10^7^
III	60 · 10^9^	1 · 10^−3^	0.1 · 10^−6^	2.4 · 10^7^

## Data Availability

The data used to support the findings of this study are available from the corresponding author upon request.
